# Supplementary material for: The role of structural connectivity on brain function through a Markov model of signal transmission
Source: PLoS One. 2025 Sep 11;20(9):e0331085. doi: 10.1371/journal.pone.0331085 (PMC12425331; doi:10.1371/journal.pone.0331085)
Supplement: S1 File — (PDF) [file pone.0331085.s001.pdf]

Supplementary Information

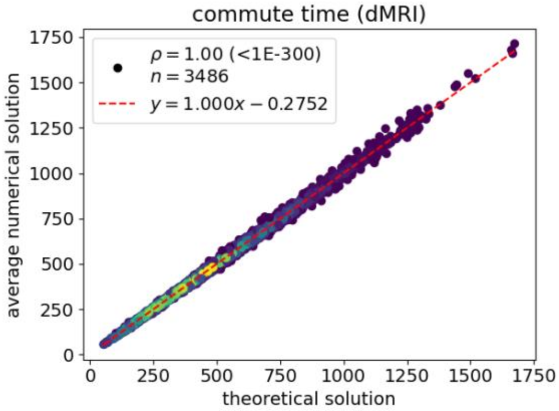

***Figure S1: Theoretical expression for commute time (Equation 6) matches numerical results**. Commute time is calculated and simulated for a dMRI-derived structure from an arbitrary UK Biobank individual (subject id: 1000366) under the Desikan-Killiany atlas (84 brain regions). We run 1,000 replicate simulations for each pair of brain regions and take the mean number of steps to compare with the theoretical solution. Data points correspond to all possible pairs of brain regions (n = 84 x 83 / 2) and are colored to capture the density of points, with brighter colors corresponding to greater density. The symbol ρ corresponds to the Spearman correlation coefficient with its p-value following in parenthesis. The dashed red-line corresponds to the best-fit line. The equation is written in the legend where y corresponds to the average numerical solution; x corresponds to the theoretical solution.*

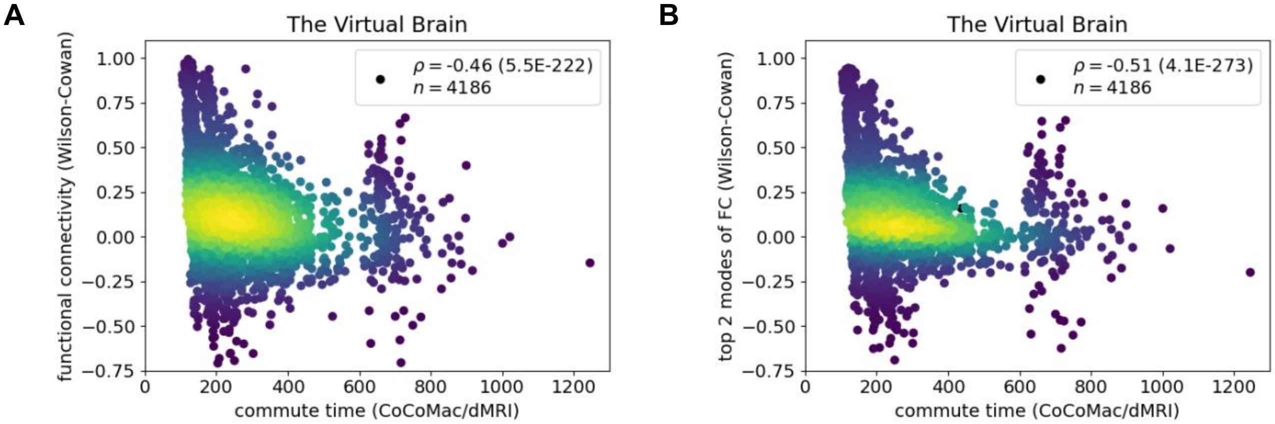

***Figure S2: Commute time captures functional connectivity in a Wilson-Cowan simulation of neurosignaling**. The Wilson-Cowan simulation is run on connectivity data merged from the macaque brain (CoCoMac* (100,101)*) and dMRI provided by The Virtual Brain* (42). *Parameter values can be found in Table S1 and are chosen such that neuronal regions demonstrate stable oscillatory dynamics. Neuronal signals are convoluted with the Balloon model* (43) *to output a fMRI BOLD-like signal. The same simulation is presented in both **A** and **B**; the difference is that only the result from the top two modes of the FC matrix is presented in **B**. For visual clarity, the x-axes are cutoff at a commute time value of 1300, however, there are a cluster of commute times at 4750 and they are included in correlation calculations and n.*

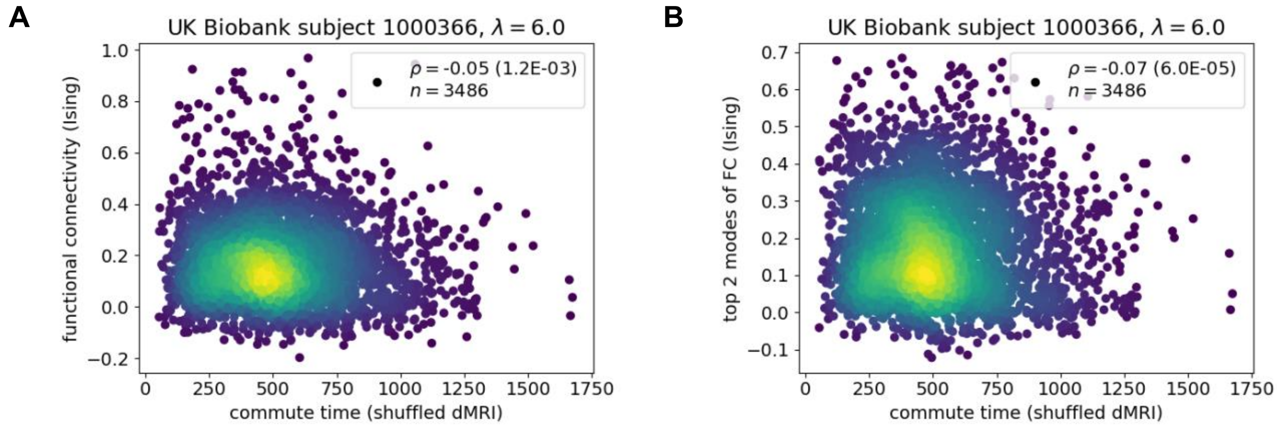

Figure S3: Shuffling the structural connectivity matrix and then simulating function leads to a poor correlation with commute time, calculated from the original structural connectivity matrix. *A and B correspond to the same plots as Figure 2A and Figure 2B for the same UK Biobank individual.*

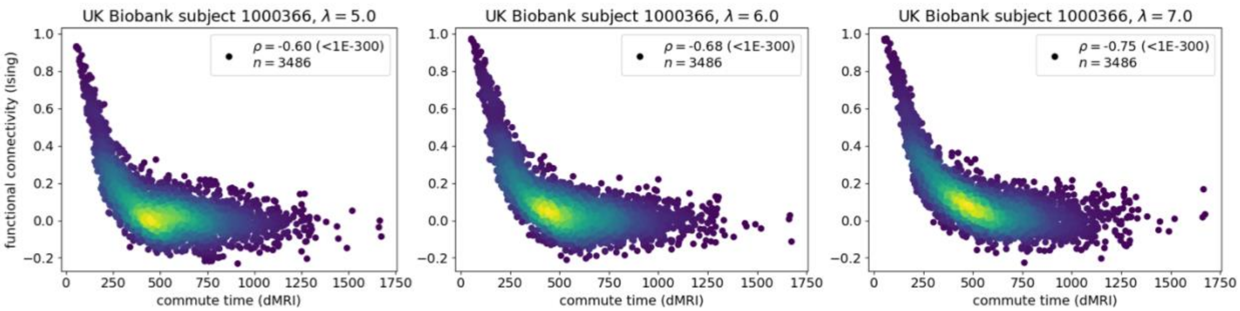

Figure S4: The commute time-functional connectivity correlation is mediated by the Ising model's coupling strength λ. *Three different Ising simulations are run on a dMRI-derived structure from the same UK Biobank individual (subject id: 1000366) for three different coupling strength values. The middle panel (λ = 6.0) is also displayed in Figure 2A.*

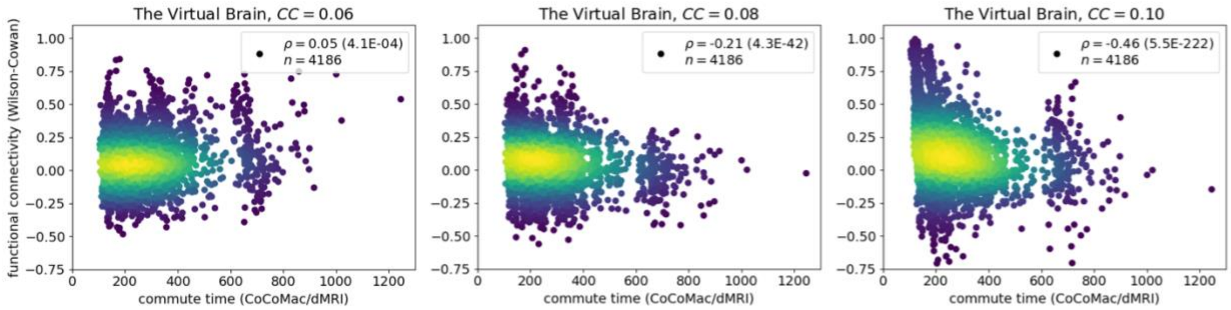

Figure S5: The commute time-functional connectivity correlation evaluated using the Wilson-Cowan model's global coupling constant (CC). *All other parameters are the same as in Figure S2 and can be found in Table S1. Three different Wilson-Cowan simulations are run on connectivity data merged from the macaque brain (CoCoMac* (100,101)*) and dMRI provided by The Virtual Brain* (42) *for three different coupling constant values. For visual clarity, the x-axes are cutoff at a commute time value of 1300, however, there are a cluster of commute times at 4750 and they are included in correlation calculations and n. The right plot (CC = 0.10) is also displayed in Figure S2A.*

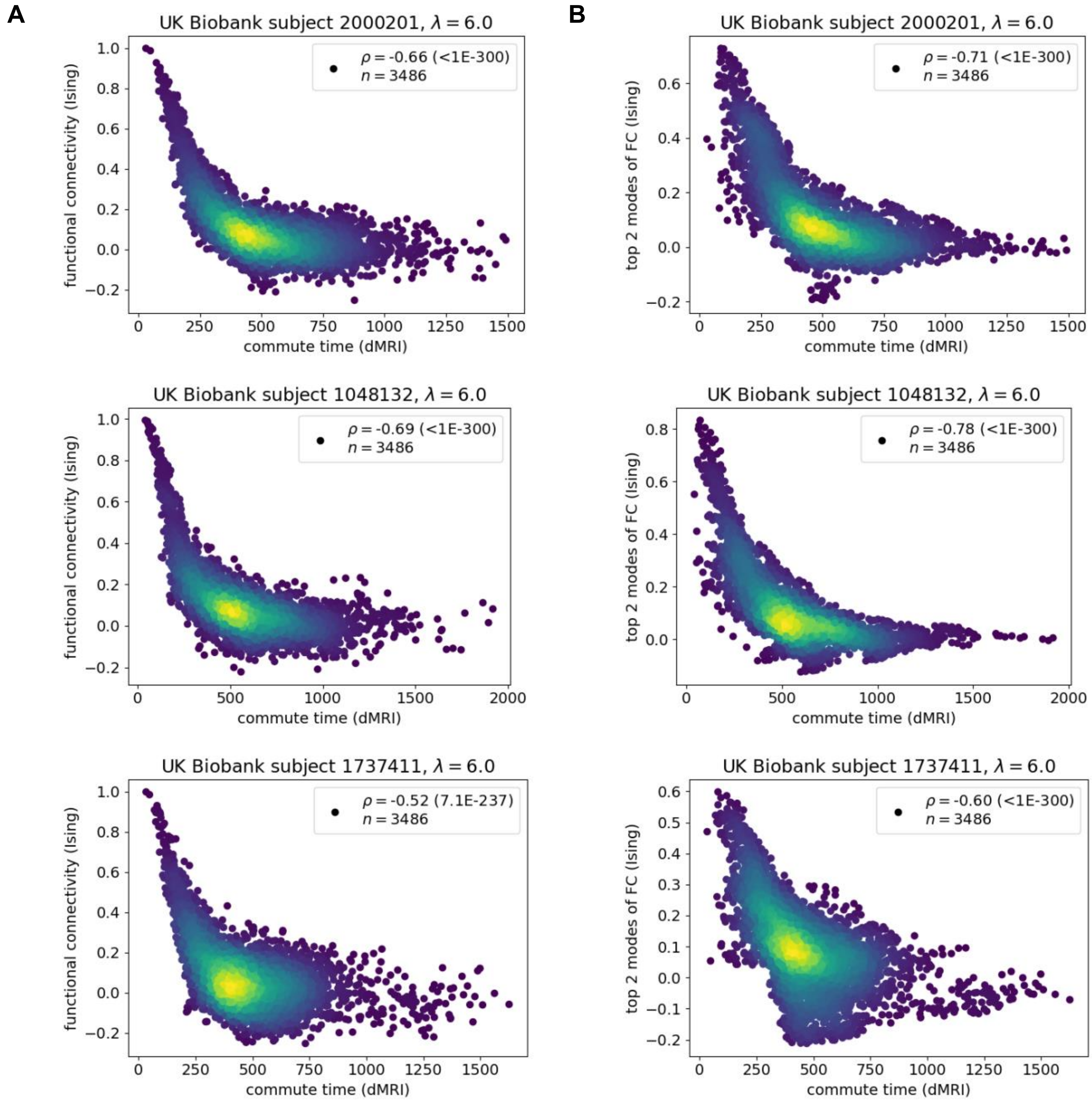

Figure S6: Commute time captures functional connectivity in mean-field Ising simulations for three different individuals' structures from the UK Biobank. *The same simulation is presented in each row of* ***A*** *and* ***B****; the difference is that only the top two modes of the FC matrix are included in* ***B****. Three sets of Ising simulations are performed on a dMRI-derived structure from three different UK Biobank individual (subject id: 2000201, 1048132, 1737411) under the Desikan-Killiany atlas (84 brain regions). The same global coupling strength parameter λ = 6.0 is employed for all individuals (Methods).*

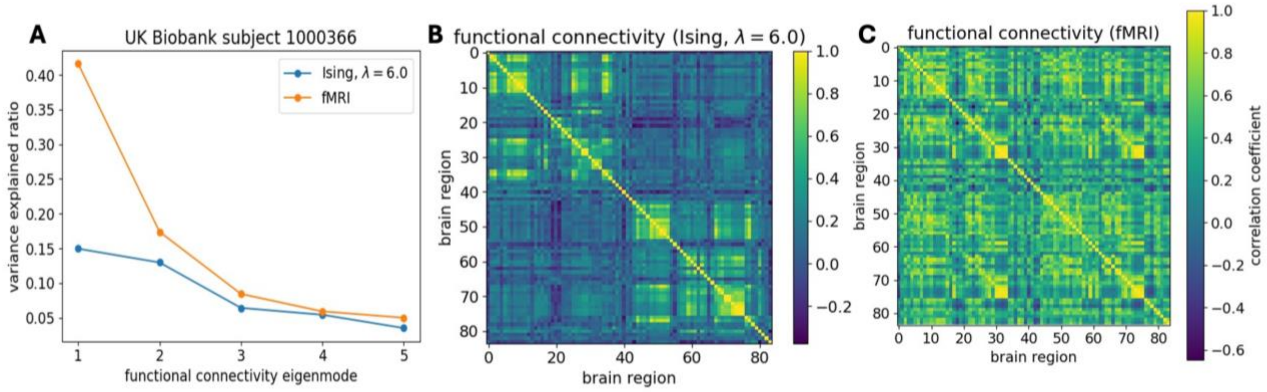

Figure S7: Comparison of the FC matrices based on Ising simulations and fMRI data. A. *The largest eigenvalues of the FC matrices from an Ising simulation on a dMRI-derived structure from subject 1000366 (blue curve) and the FC matrix from fMRI data of the same subject (orange curve). A coupling strength parameter λ = 6.0 is adopted under the Desikan-Killiany atlas (84 brain regions). FCs are the same as those presented in Figure 2A and in Figure 3A, respectively.* ***B-C.*** *The FC matrix derived from the Ising simulation with a coupling strength parameter λ = 6.0 on subject 1000366's dMRI structure under the Desikan-Killiany atlas (**B**) is qualitatively different than that of the FC matrix derived from fMRI data on the same individual under the same atlas (**C**).*

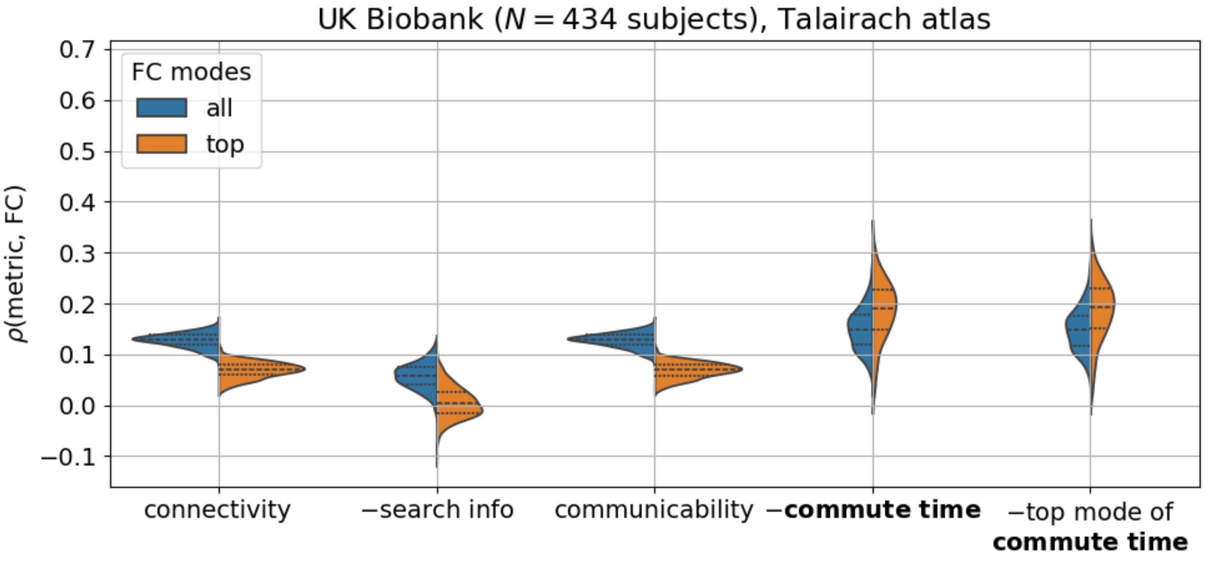

Figure S8: Commute time outperforms other structure-based metrics at predicting functional connectivities regardless of the brain atlas across the UK Biobank. *fMRI and dMRI data are processed according to the Talairach atlas (727 brain regions on average (Methods)). Corresponding Kolmogorov-Smirnov pairwise tests between metrics are summarized in Table S6.*

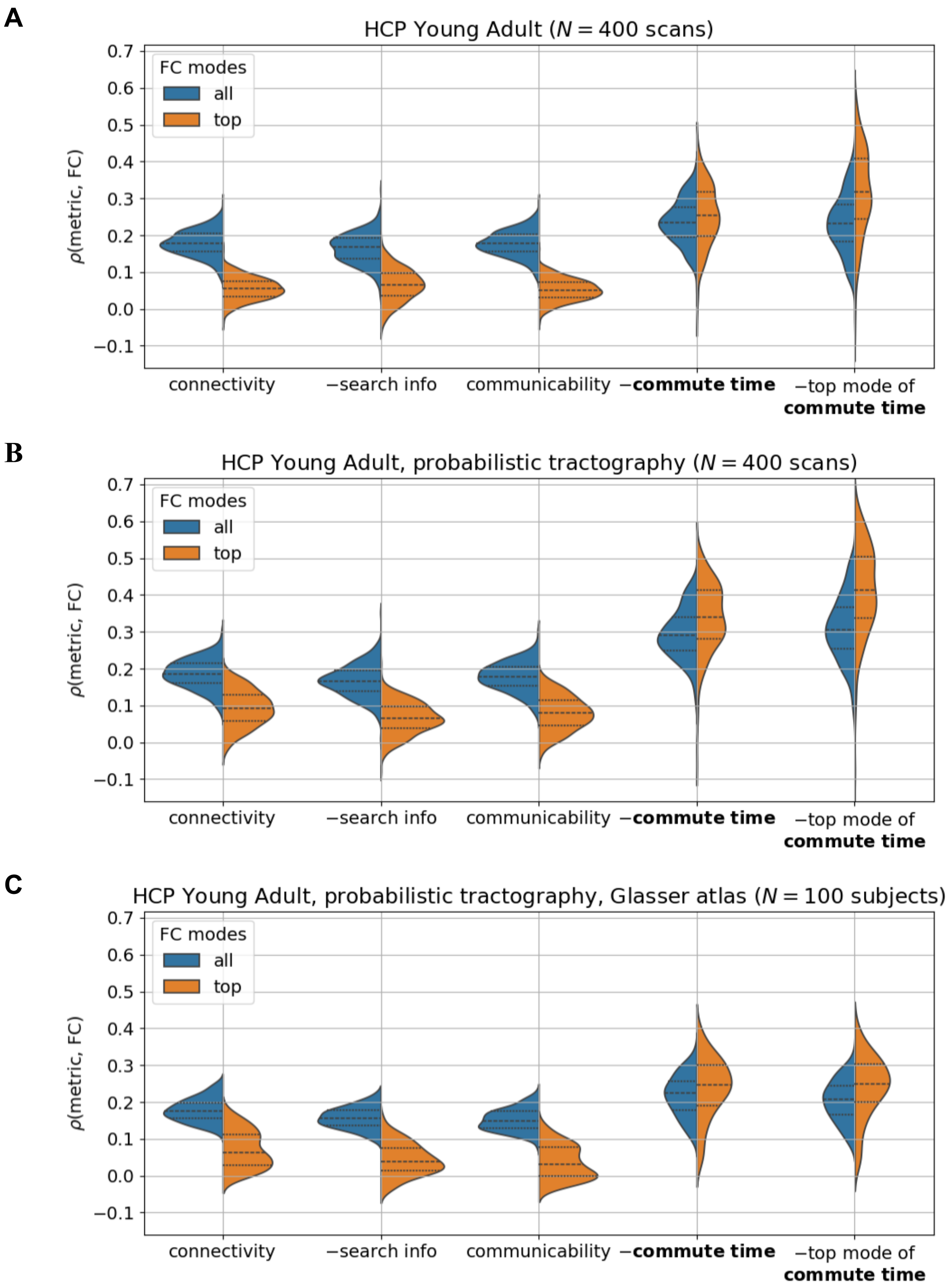

Figure S9: Commute time outperforms other structure-based metrics at predicting FCs for the Human Connectome Project (HCP) Young Adult dataset*. N corresponds to the number of brain scans considered from HCP Young Adult. Each subject had 4 brain scans and we consider individuals from the 100 Unrelated Subjects subset. fMRI and dMRI data are processed according to the Desikan-Killiany atlas. Commute time outperforms regardless of the tractography method (****A**** is deterministic, ****B-C**** are probabilistic) or the atlas (****A-B**** is the Desikan-Killiany atlas (84 brain regions), ****C**** is the Glasser atlas (360 brain regions)* (102)*). Results presented in ****C**** are taken from public data made available from another study* (16).

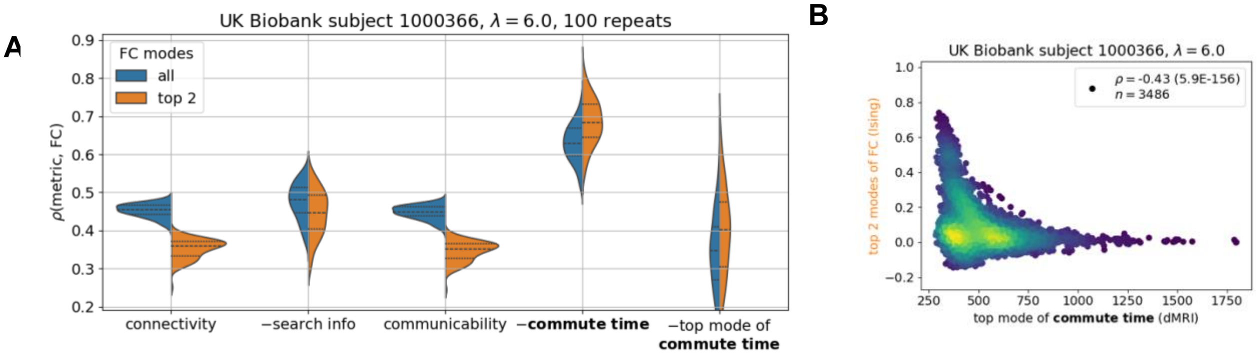

Figure S10: Top mode of commute time performs poorly for Ising simulations because function is entirely determined by the inputted structure. A. *is a reproduction of* ***Figure 2A*** *under the same y-axis limits with an added column at the right corresponding to the top mode of commute time.* ***B.*** *demonstrates a commute time-FC scatterplot across brain pairs when considering their respective top principal components (same simulation run as shown in* ***Figure 2A-B****). dMRI data is processed according to the Desikan-Killiany atlas.*

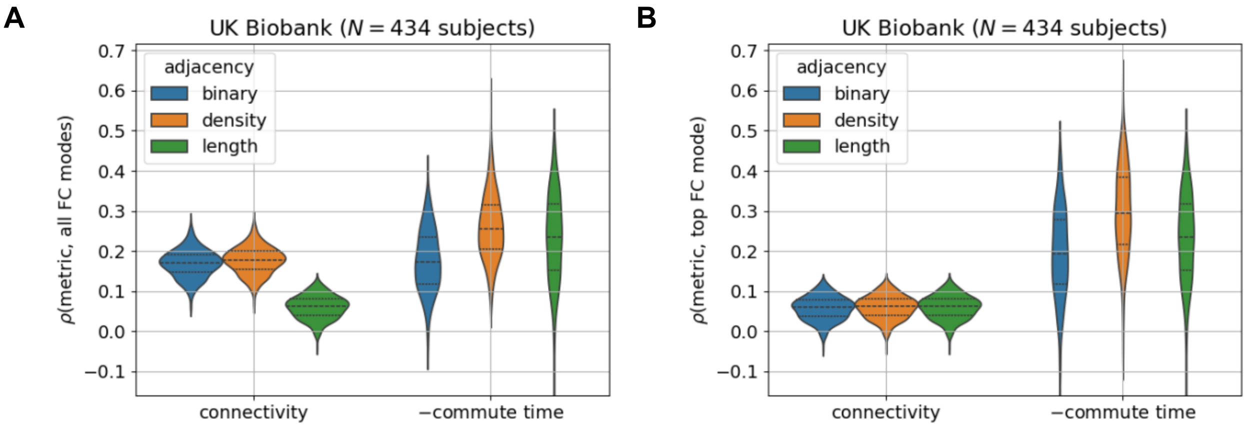

Figure S11: Commute time calculated based on the number of tracts (also called density) outperforms other alternative definitions for a brain network's adjacency matrix. A. *demonstrates results when considering all FC modes;* ***B.*** *only considers the top FC mode when calculating the commute time-FC correlation. Note that the density distributions (shown in orange in A and B) are the same as those shown in Figure 3C. fMRI and dMRI data are processed according to the Desikan-Killiany atlas.*

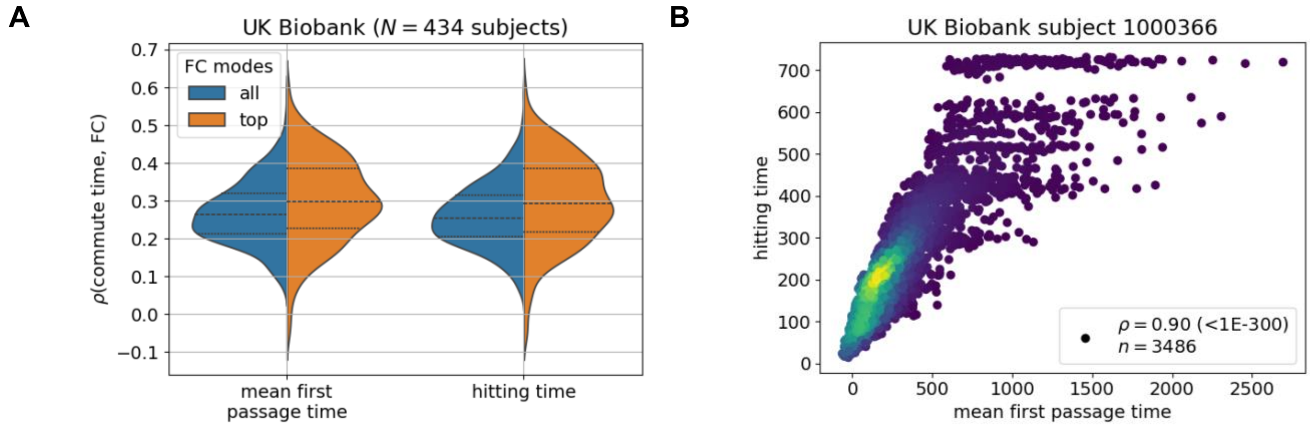

Figure S12: Commute time calculated via hitting time or mean first passage time yield similar results. A. *demonstrates correlation results across all considered UK Biobank subjects;* ***B.*** *demonstrates how closely hitting times and mean first passage times correlate for an arbitrary UK Biobank subject across all possible pairs of its brain regions. fMRI and dMRI data are processed according to the Desikan-Killiany atlas.*

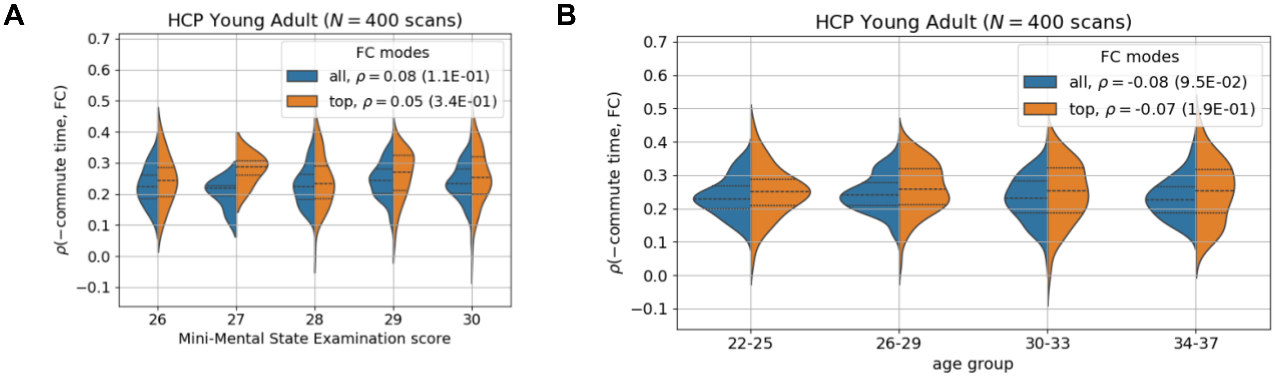

Figure S13: Minimal differences are seen between brain scans from different Mini-Mental State Examination scores (A), and different age groups (B) in the HCP Young Adult dataset. *fMRI and dMRI data are processed according to the Desikan-Killiany atlas.*

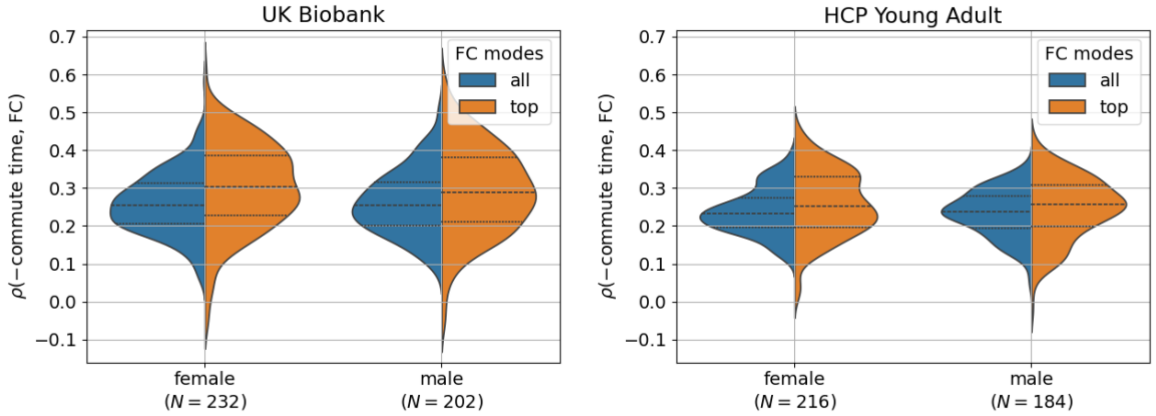

Figure S14: Minimal differences are seen in the commute time-FC correlation across brain scans from different sexes in the UK Biobank and HCP Young Adult datasets. *N corresponds to the number of brain scans considered for that particular group. fMRI and dMRI data are processed according to the Desikan-Killiany atlas.*

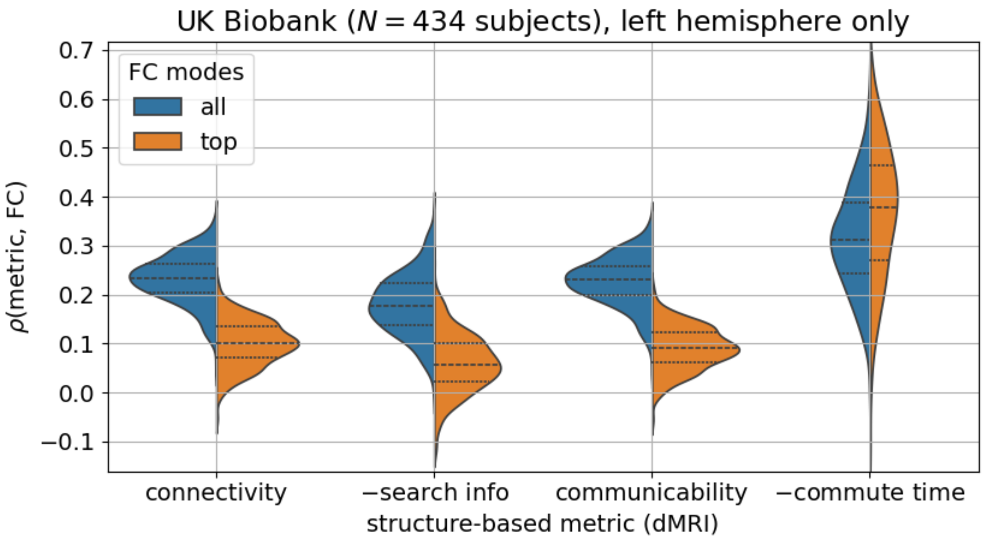

Figure S15: Commute time outperforms other structure-based metrics at predicting functional connectivities (FCs), even when only analyzing intra-hemisphere connections from the left hemisphere. *Corresponding Kolmogorov-Smirnov pairwise tests between metrics are summarized in Table S7. fMRI and dMRI data are processed according to the Desikan-Killiany atlas.*

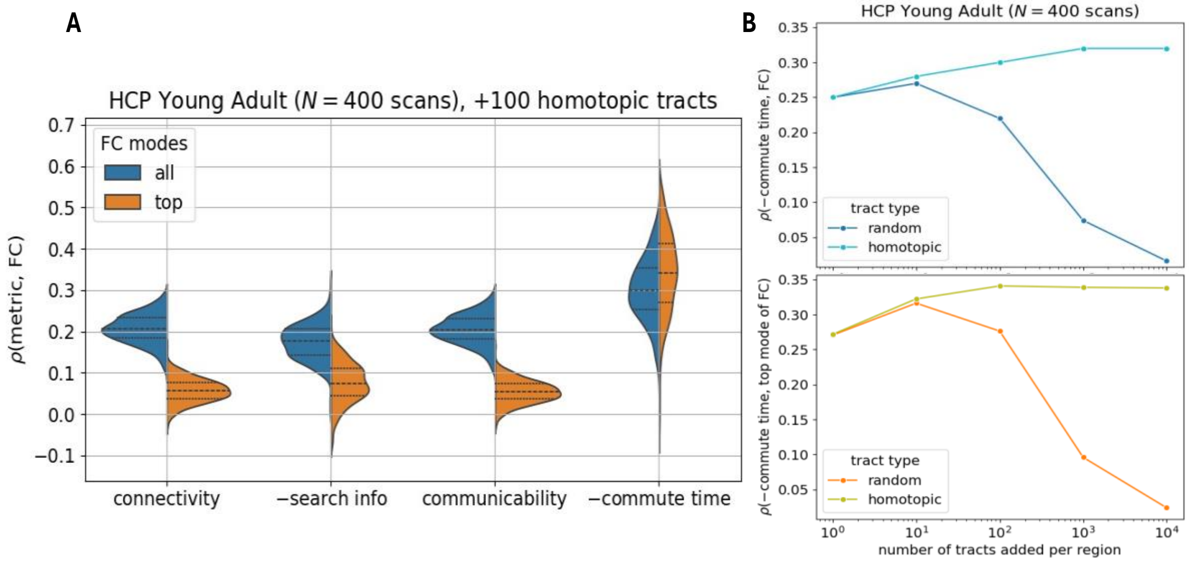

Figure S16: Effect of adding 100 homotopic connections per region between the two same regions across the brain hemispheres. A. *An improvement in structure-function correlations is observed, with commute time outperforming other structure-based metrics.* ***B.*** *White matter tracts added between homotopic regions improve the average commute time-FC correlation, especially compared to randomly added connections. The full distribution across all HCP Young Adult brain scans from the 100 Unrelated Subjects subset is shown for 100 added homotopic connections. Assessments are made for every order of magnitude up to 10,000 added white matter tracts. The entirety of the FC is considered in the top panel; only the top mode of the FC matrix is considered the bottom panel. fMRI and dMRI data are processed according to the Desikan-Killiany atlas.*

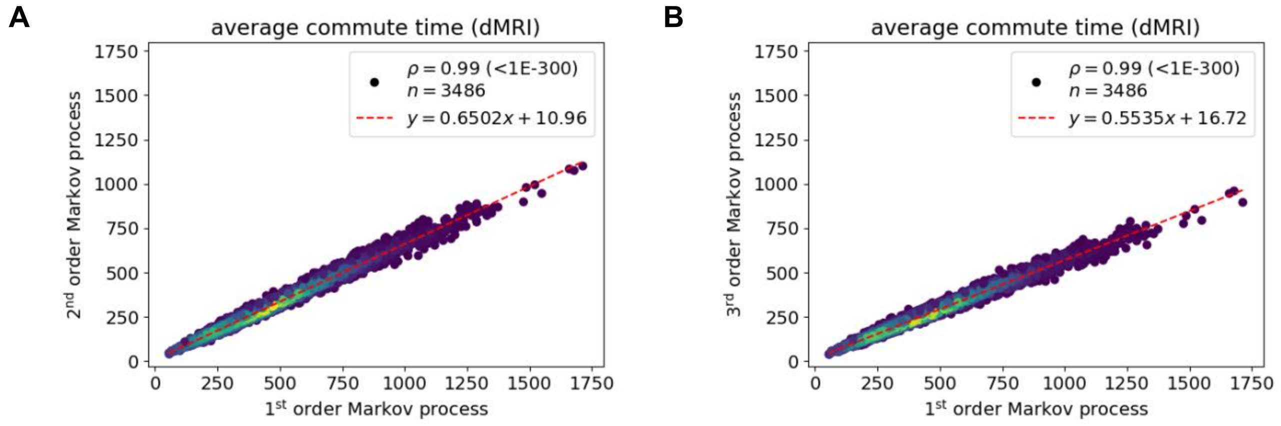

***Figure S17: Numerically calculating commute time with memory (2nd order and 3rd order Markov processes) strongly correlates with commute time without memory (1st order Markov process)**. The first order Markov process corresponds to the same simulation as in Figure S1 and has been simply called commute time throughout the text and SI. Second order and third order Markov processes are calculated by tweaking the 1st order simulation. For 2nd order, transition probabilities going back to the previous node along its path are set to zero. For example, if we have a network A-B-C, the following path is **not allowed** in a 2nd order Markov process: A→B**→A**→B→C. The subsequent rule is extended for a 3rd order Markov process for which the two previous nodes along its path are 'remembered.' All simulations are run on a dMRI-derived structure from an arbitrary UK Biobank individual (subject id: 1000366) under the Desikan-Killiany atlas. We run 1,000 replicate simulations for each pair of brain regions and plot the mean number of steps.*

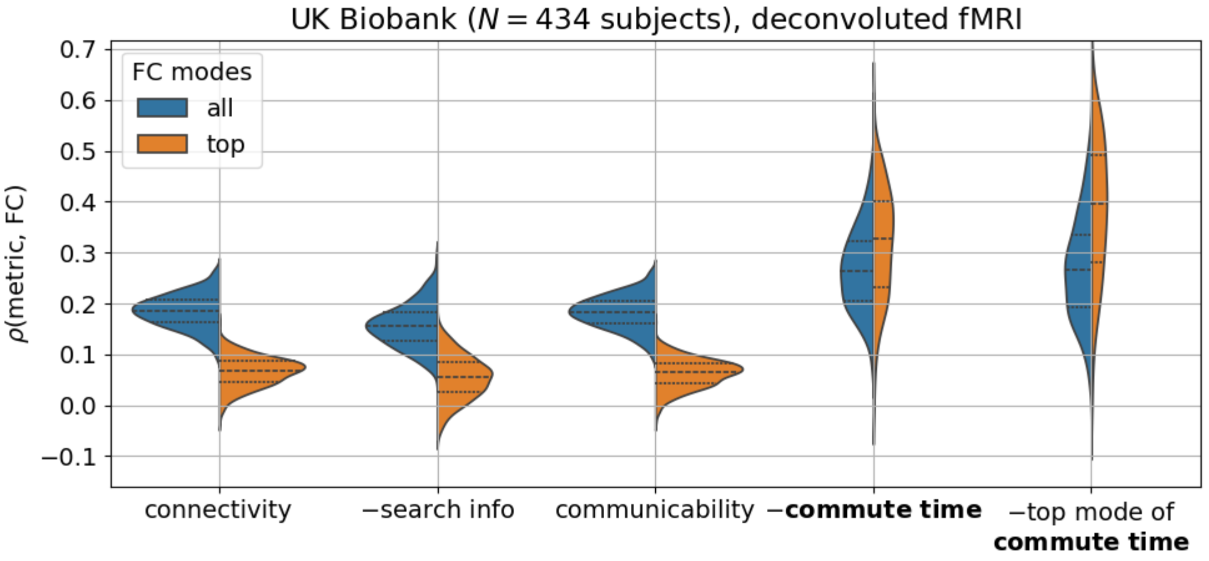

***Figure S18: Commute time outperforms other structure-based metrics at predicting functional connectivities (FCs), when FCs are calculated based on deconvoluted fMRI BOLD signals.** We used the rsHRF toolbox to deconvolve processed fMRI images that have not yet been parcellated with the gamma estimation method* (75).

Parameter	Description	Value
	Population time constants	
r_e	Excitatory refractory period [ms]	1 ms
r_i	Inhibitory refractory period [ms]	1 ms
k_e	Maximum value of the excitatory response function	1
k_i	Maximum value of the inhibitory response function	1
τ_e	Excitatory population, membrane time-constant [ms]	8 ms
τ_i	Inhibitory population, membrane time-constant [ms]	8 ms
	Local network connectivity parameters	
c_{ee}	Excitatory to excitatory coupling coefficient	15.8
c_{ei}	Inhibitory to excitatory coupling coefficient	15.6
c_{ie}	Excitatory to inhibitory coupling coefficient	30
c_{ii}	Inhibitory to inhibitory coupling coefficient	6
α_e	Balance parameter between excitatory and inhibitory masses	1
α_i	Balance parameter between excitatory and inhibitory masses	1
	Activation function parameters	
a_e	The slope parameter for the excitatory response function	1
a_i	The slope parameter for the inhibitory response function	1
b_e	Position of the maximum slope of the excitatory sigmoid function	0
b_i	Position of the maximum slope of the inhibitory sigmoid function	0
c_e	The amplitude parameter for the excitatory response function	1
c_i	The amplitude parameter for the inhibitory response function	1
ϑ_e	Excitatory threshold	0
ϑ_i	Inhibitory threshold	0
	External perturbation parameters	
P	External stimulus to the excitatory population	-3.575
Q	External stimulus to the inhibitory population	-7.4
	Global Connectivity parameters	
CV	Conduction velocity between brain regions	3.0
CC	Coupling constant between brain regions	0.1

Table S1: Wilson-Cowan model parameter descriptions and values for Figure S2. Descriptions are adapted from Tables 10 and 11 of Sanz-Leon et al. (42).

KS \\ p-value	connectivity	search info	communicability	commute time
connectivity	\\	1.0E-11 | 3.1E-31	0.11 | 0.21	2.2E-59 | 2.2E-59
search info	0.50 | 0.79	\\	3.4E-13 | 3.9E-33	2.6E-44 | 4.4E-55
communicability	0.17 | 0.15	0.53 | 0.81	\\	2.2E-59 | 2.2E-59
commute time	1.0 | 1.0	0.91 | 0.98	1.0 | 1.0	\\

Table S2: Summary statistics of Kolmogorov-Smirnov (KS) pairwise tests for distributions presented in Figure 2C when including all modes (left of the vertical line |) and only the top mode (right of |) of the functional connectivity matrix from Ising simulations on UK Biobank (subject id: 1000366) with a coupling strength parameter λ = 6. KS test statistics are entered in the bottom left of the table; corresponding p-values are in the top right.

KS \\ p-value	connectivity	search info	communicability	commute time
connectivity	\\	4.0E-18 | 5.1E-07	0.89 | 0.33	2.3E-70 | 1.1E-216
search info	0.30 | 0.19	\\	5.2E-16 | 2.4E-5	2.6E-84 | 3.3E-203
communicability	0.040 | 0.065	0.29 | 0.16	\\	1.5E-71 | 2.9E-218
commute time	0.59 | 0.95	0.64 | 0.93	0.59 | 0.95	\\

Table S3: Summary statistics of Kolmogorov-Smirnov (KS) pairwise tests for distributions presented in Figure 3C when including all modes (left of the vertical line |) and only the top mode (right of |) of the functional connectivity matrix from fMRI data of 434 UK Biobank individuals. KS test statistics are entered in the bottom left of the table; corresponding p-values are in the top right.

	Disease name	ICD-10	total
**Mental disorder**	Psychotic disorder due to multiple drug use	F19.5	1
	Acute and transient psychotic disorder, unspecified	F23.9	1
	Anxiety Disorder, Unspecified	F41.9	1
	Sexual arousal disorders	F52.2	1
**Nerve disorder**	Epilepsy, unspecified	G40.9	3
	Transient cerebral ischemic attack, unspecified	G45.9	2
	Sleep apnea	G47.3	2
	Other sleep disorders	G47.8	1
	Carpal Tunnel Syndrome	G56.0	3
	Lesion of ulnar nerve	G56.2	1
	Lesion of the sciatic nerve	G57.0	1

Lesion of the plantar nerve	G57.6	1
Other specified mononeuropathies of lower limb	G57.8	1

Table S4: List of disorders considered in our analyses in Figure 4A,C. Entries in the ICD-10 column corresponds to the International Classification of Diseases-10 coding system. The total column corresponds to the number of considered UK Biobank individuals diagnosed with the respective disorder for that row.

KS \\ p-value	mental disorder	nerve disorder	healthy
mental disorder	\\	0.15 | 0.35	0.12 | 0.27
nerve disorder	0.6 | 0.48	\\	0.78 | 0.32
healthy	0.55 | 0.46	0.16 | 0.24	\\

Table S5: Summary statistics of Kolmogorov-Smirnov (KS) pairwise tests for distributions presented in Figure 4A when including all modes (left of the vertical line |) and only the top mode (right of |) of the functional connectivity matrix from fMRI data of 434 UK Biobank individuals. KS test statistics are entered in the bottom left of the table; corresponding p-values are in the top right.

KS \\ p-value	connectivity	search info	communicability	commute time
connectivity	\\	3.3E-203 | 1.2E-152	1.0 | 1.0	2.9E-43 | 1.7E-184
search info	0.93 | 0.83	\\	1.2E-204 | 1.3E-151	8.5E-168 | 4.4E-206
communicability	0.014 | 0.028	0.93 | 0.83	\\	2.9E-43 | 1.7E-184
commute time	0.47 | 0.90	0.86 | 0.93	0.47 | 0.90	\\

Table S6: Summary statistics of Kolmogorov-Smirnov (KS) pairwise tests for distributions presented in Figure S8 when including all modes (left of the vertical line |) and only the top mode (right of |) of the functional connectivity matrix from fMRI data under the Talairach atlas of 434 UK Biobank individuals. KS test statistics are entered in the bottom left of the table; corresponding p-values are in the top right.

KS \\ p-value	connectivity	search info	communicability	commute time
connectivity	\\	1.1E-32 | 1.5E-21	0.47 | 0.0098	1.7E-45 | 1.4E-173
search info	0.41 | 0.33	\\	5.5E-28 | 2.9E-16	2.8E-78 | 6.2E-176
communicability	0.058 | 0.11	0.38 | 0.29	\\	2.9E-48 | 3.0E-183
commute time	0.48 | 0.88	0.62 | 0.88	0.49 | 0.89	\\

Table S7: Summary statistics of Kolmogorov-Smirnov (KS) pairwise tests for distributions presented in Figure S15 when including all modes (left of the vertical line |) and only the top mode (right of |) of the functional connectivity matrix from fMRI data for only left hemisphere regions of 434 UK Biobank individuals. KS test statistics are entered in the bottom left of the table; corresponding p-values are in the top right.

Dataset	age range	⟨age⟩ ± std(age)	sex
**UK Biobank**	46-79	62.6 ± 7.4	232F / 202M
**HCP Young Adult**	22-36	29.1 ± 3.7	54F / 46M

Table S8: Demographic information of the individuals considered in this paper. Ages are in units of years. The average age ⟨age⟩ *and standard deviation (std) are presented.*

Dataset	field	repetition	echo	flip angle	voxel size	total time
**UK Biobank**	3T	735 ms	39 ms	52°	2.4x2.4x2.4 mm^3	490
**HCP Young**	3T	720 ms	33.1 ms	52°	2x2x2 mm^3	1200

Table S9: Functional MRI acquisition parameters of the datasets.

S1 Text. Commute time derivation

We expand upon the derivation presented in the Methods by illustrating how applying Greville's formula twice takes us from Equation 10 to Equation 11. If $\boldsymbol{A}_k$ is a *m* x *k* matrix, Greville's formula states that by appending an additional column a_{k+1}, the inverse of the resulting matrix ($\boldsymbol{A}_{k+1}^{\dagger}$) can be expressed as (84,85),

Equation 15

$$\boldsymbol{A}_{k+1}^{\dagger} = [\boldsymbol{A}_k, a_{k+1}]^{\dagger} = \begin{bmatrix} \boldsymbol{A}_k^{\dagger} - d_k b_k \\ b_k \end{bmatrix}$$

The symbols d_k and b_k are defined as,

Equation 16

$$d_k = \boldsymbol{A}_k^{\dagger} a_{k+1},\ c_k = a_{k+1} - \boldsymbol{A}_k d_k$$
$$b_k = \begin{cases} c_k^{\dagger}, & \text{if } c_k \neq 0 \\ \left(1 + d_k^T d_k\right)^{-1} d_k^T \boldsymbol{A}_k^{\dagger}, & \text{if } c_k = 0 \end{cases}$$

Note that the symbol † is used to denote the matrix inverse rather than $^{-1}$ to clarify that the pseudoinverse is taken, rather than the traditional inverse. We need to take the pseudoinverse because $\boldsymbol{\Gamma}$ has a zero eigenvalue and $\widetilde{\boldsymbol{\Gamma}}$ is not square. For simplicity, we refer to the pseudoinverse as $^{-1}$ throughout the main text and the rest of this supplementary text.

With Greville's formula, we can express $\widehat{\boldsymbol{\Gamma}}^{-1}$ in terms of $\boldsymbol{\Gamma}^{-1}$ as follows. First, we define $\widetilde{\boldsymbol{\Gamma}}$ as an (*n*-1) x *n* matrix.

Equation 17

$$\boldsymbol{\Gamma} = \begin{bmatrix} \widetilde{\boldsymbol{\Gamma}} \\ r^T \end{bmatrix}$$

It can be shown that the row vector $r^T = -\widehat{\mathbf{1}}^T \widetilde{\boldsymbol{\Gamma}}$ ensures the condition that the sum of rows of the Laplacian matrix is equal to zero ($\mathbf{1}^T \boldsymbol{\Gamma} = \mathbf{0}$).

To connect $\widetilde{\boldsymbol{\Gamma}}$ to $\widehat{\boldsymbol{\Gamma}}$, we have the following relationship:
Equation 18

$$\widetilde{\boldsymbol{\Gamma}} = [\widehat{\boldsymbol{\Gamma}}, s]$$

It can be shown that the vector $s = -\widehat{\boldsymbol{\Gamma}}\widehat{\mathbf{1}}$ ensures that the sum of columns of the Laplacian matrix is equal to 0 ($\boldsymbol{\Gamma}\mathbf{1} = \mathbf{0}^T$).

We are now ready to apply Greville's formula. We first calculate the pseudoinverse of $\widetilde{\boldsymbol{\Gamma}}$,
Equation 19

$$\widetilde{\boldsymbol{\Gamma}}^{-1} = \begin{bmatrix} \widehat{\boldsymbol{\Gamma}}^{-1} + \widehat{\mathbf{1}} b_k \\ b_k \end{bmatrix}$$

We do not need to directly evaluate b_k from Equation 16. Instead, we notice that b_k must be equal to the removed j^{th} row in $\widehat{\boldsymbol{\Gamma}}$ that is present in $\widetilde{\boldsymbol{\Gamma}}$.
Equation 20

$$\widetilde{\boldsymbol{\Gamma}}^{-1} = \begin{bmatrix} \widehat{\boldsymbol{\Gamma}}^{-1} \\ 0 \end{bmatrix} + \begin{bmatrix} \widehat{\mathbf{1}} b_k \\ b_k \end{bmatrix}$$
$$\rightarrow b_k = \left[\widetilde{\boldsymbol{\Gamma}}^{-1}\right]_j$$

In component form, Equation 20 is equivalent to:
Equation 21

$$\left[\widetilde{\boldsymbol{\Gamma}}^{-1}\right]_{lm} = \left[\widehat{\boldsymbol{\Gamma}}^{-1}\right]_{lm} + \left[\widetilde{\boldsymbol{\Gamma}}^{-1}\right]_{jm}$$
$$\rightarrow \left[\widehat{\boldsymbol{\Gamma}}^{-1}\right]_{lm} = \left[\widetilde{\boldsymbol{\Gamma}}^{-1}\right]_{lm} - \left[\widetilde{\boldsymbol{\Gamma}}^{-1}\right]_{jm}$$

We can perform the same steps as in Equation 19 and Equation 20 to express $\widetilde{\boldsymbol{\Gamma}}^{-1}$ in terms of $\boldsymbol{\Gamma}^{-1}$,
Equation 22

$$\left[\widetilde{\boldsymbol{\Gamma}}^{-1}\right]_{no} = [\boldsymbol{\Gamma}^{-1}]_{no} - [\boldsymbol{\Gamma}^{-1}]_{jn}$$

Inserting Equation 22 into Equation 21, we finally have our expression for $\widehat{\boldsymbol{\Gamma}}^{-1}$ in terms of $\boldsymbol{\Gamma}^{-1}$.
Equation 23

$$\left[\widehat{\boldsymbol{\Gamma}}^{-1}\right]_{lm} = [\boldsymbol{\Gamma}^{-1}]_{lm} - [\boldsymbol{\Gamma}^{-1}]_{jl} - [\boldsymbol{\Gamma}^{-1}]_{jn} + [\boldsymbol{\Gamma}^{-1}]_{jj}$$

Equation 23 is inserted in Equation 10 to obtain Equation 11.

S2 Text. Mean first passage time vs. hitting time

The main difference between the two diffusion-based metrics is in evaluating Equation 7. For hitting time, we rewrite Equation 7 in terms of truncated matrices in Equation 8 to satisfy the condition in the summation that $k \neq j$. For mean first passage time, we keep the full matrix and subtract off the mean recurrence time of node i, r_i (103).

Equation 24

$$r_i = 1 + \sum_{k}^{N} M_{ik} H_{kj}$$

Mean recurrence is the amount of time it takes to return to the initial state. Note that r_i is different than commute time, which considers the time it takes to return to the initial state *i* after first reaching some destination node *j*.

We can now rewrite Equation 7 in terms of the **R** matrix, where non-diagonal elements are equal to zero and diagonal elements $\mathbf{R}_{ii} = r_i$.

Equation 25

$$\boldsymbol{H} = \mathbf{1} + \boldsymbol{MH} - \mathbf{R}$$

Note that unlike Equation 8, we write the hitting time in terms of a *N*x*N* matrix, rather than a row vector in Equation 25. Also note that we have yet to introduce mean first passage time in our expression in Equation 25. So far, Equation 25 is exact and still corresponds to hitting time. However, we introduce the notation **F** representing the mean first passage time to replace ***H*** given the following approximations to solve for **F** in Equation 27.

Equation 26

$$\mathbf{F} = \mathbf{1} + \boldsymbol{M}\mathbf{F} - \mathbf{R}$$

Using the fundamental matrix **Z** and assuming that it converges when a large amount of steps are taken (large time limit) (103), it can be shown that the mean first passage time F_{ij} from node *i* to *j* is:

Equation 27

$$F_{ij} = \frac{Z_{jj} - Z_{ij}}{w_j}$$

The term w_j corresponds to the *j*th component of the fixed probability vector for the transition matrix ***M***. The fundamental matrix **Z** is defined as,

Equation 28

$$\mathbf{Z} = (\mathbf{1} - \boldsymbol{M} + \mathbf{W})^{-1}$$

For much more details on how to derive Equation 27, please refer to reference (103)**.**
