# Supplementary material for: The role of structural connectivity on brain function through a Markov model of signal transmission
Source: PLoS One. 2025 Sep 11;20(9):e0331085. doi: 10.1371/journal.pone.0331085 (PMC12425331; doi:10.1371/journal.pone.0331085)
Supplement: Attachment — Submitted filename: Response to Reviewers.docx [file pone.0331085.s003.docx]

**Response to Reviewers**:

Thank you for thoroughly reviewing our manuscript and taking the time to provide constructive suggestions and comments. Considering and incorporating them has greatly enhanced the quality of the manuscript. We proceed to respond to each reviewers’ comments point-by-point and indicate the corresponding changes in the main text in purple. Excerpts from the revised manuscript are indented and written in smaller font below.

Here is a brief summary of the changes made:

1. Clarified the motivations of commute time and how it differentiates from other communication metrics in the Introduction. Clarified how commute time is calculated and considered alternative adjacency matrices, such as length (Figure S11).
2. Considered another communication metric called mean first passage time (Figure S12 and Text S2).
3. Highlighted the importance of extrasynaptic communication as future work to better bridge structure to function in the Discussion.
4. Explicitly list the names of the diseases included in Figure 4 in Table S4.

*Reviewer #1: RECOMMENDATIONS:

Physical vs. topological distance: It was not immediately clear from the text whether the commute time is computed using physical distance or graph-theoretic distance (i.e. number of edges traversed). This is an important distinction. For instance, a long-range callosal axon connecting opposite hemispheres likely differs substantially in transmission properties from a short-range local tract, even if both are represented by a single edge in the connectivity graph. I encourage the authors to clarify this point and, if applicable, to discuss the implications of using graph distance versus physical distance, especially in light of the heterogeneity in axon length, conduction velocity, and energy cost.*

Results are based on the graph-theoretic distance, whilst taking into account the number of streamlines (weighted edge). We apologize for the confusion and added text to the first paragraph of the Results to clarify this point.

It is certainly plausible that physical distance should play an important in neurosignaling. We did additional analyses and found that commute time calculated based on tract distances exhibit poorer performance compared to those presented in the main text based on streamline counts (Figure S11). Experimentally found variations in conduction velocities such that signals along edges reach their destination at the same time could explain this observation. Results from this analysis are presented in the “Commute time better captures brain function” subsection of the Results:

Throughout the main text, commute time is calculated based on the number of tracts between brain regions. However, we could adopt alternative adjacency matrices in Equation 6 to calculate the commute times based on different perspectives of brain network topology. In Figure S11, we present the commute time results when calculated with a binary representation of the adjacency matrix (*blue distributions*) or a weighted connectivity matrix based on tract lengths (*green distributions*). Commute time calculated based on the number of tracts, also called density (*orange distributions*), outperforms both of these alternative adjacency definitions. A possible explanation for the relatively lower performance of tract lengths compared to densities could be the adjustment of conduction velocities of longer tracts such that signals reach their destinations at similar times regardless of their length (49).

*We are also including here the newly generated Supplementary figure (Figure S11) for the reviewer’s convenience:*

| **A** |  | **B** |  |
| --- | --- | --- | --- |
|  |  |  |  |

**Figure S11: Commute time calculated based on the number of tracts (also called density) outperforms other alternative definitions for a brain network’s adjacency matrix. A.** demonstrates results when considering all FC modes; **B.** only considers the top FC mode when calculating the commute time-FC correlation. Note that the density distributions (shown in orange in A and B) are the same as those shown in Figure 3C. fMRI and dMRI data are processed according to the Desikan-Killiany atlas.

Energy constraints may certainly play a role in network architecture given that the brain consumes ten times more of the body’s metabolic energy as expected by its mass. However, it is not immediately clear how energy constraints directly relate to the structure-function relationship in our study.  *Consideration of extrasynaptic communication: One possible reason why structural connectivity often fails to fully explain functional connectivity is that communication in the brain is not limited to direct axonal or synaptic transmission. There is growing evidence for extrasynaptic or volume transmission via neuromodulatory systems such as monoamines and neuropeptides. These systems can exert widespread influence, bypassing classical synaptic networks. Although difficult to quantify in current human neuroimaging, this possibility should be acknowledged. The authors are encouraged to discuss such alternative modes of signalling and their potential to account for observed discrepancies between structural and functional connectivity. Recommended references:

Bentley et al. (2016), The multilayer connectome of C. elegans, PLOS Computational Biology.* [*https://doi.org/10.1371/journal.pcbi.1005283*](https://doi.org/10.1371/journal.pcbi.1005283) *Ripoll-Sánchez et al. (2023), The neuropeptidergic connectome of C. elegans. Neuron.* [*https://doi.org/10.1016/j.neuron.2023.09.043*](https://doi.org/10.1016/j.neuron.2023.09.043) *While these studies are in C. elegans, they exemplify the importance of considering multiple modes of communication in connectomic analyses.*

Thank you for sharing these references. We have now added an additional sentence highlighting the importance of extrasynaptic communication and its presence in *C. elegans.* In the last paragraph of the Discussion, we had previously mentioned in passing the potential importance of neurotransmitter expression in linking brain structure to function. However, we have now emphasized more explicitly the significance of extrasynaptic signaling (and cited the above two studies) as recommended. Below is the newly added text:

The importance of extrasynaptic neurotransmission, and in particular the modulatory effects of monoaminergic and neuropeptidergic signaling on the synaptic connectome, has been highlighted in multilayered mappings of the C. elegans connectome (82,83). These studies illustrate the rich environment underlying neurosignaling beyond axonal transmission. The universal theme in biology that structure determines function may require a more encompassing definition of ‘structure’ to meaningfully apply to the brain.

*Line 66–70: Clarification of communicability. The brief description of communicability may leave some readers unfamiliar with the metric uncertain about its mechanics and implications. Please elaborate.*

We apologize for the confusion. We have presented a clearer introduction to communicability and highlighted its differences from commute time. Mathematical differences are now fully delegated to the Methods section. *Line 483 onwards: Notation. The notation used for mean-square fluctuations appears to use less-than/greater-than symbols, which could be misinterpreted as relational operators. If the authors intended to denote averaging, they should replace these with angled brackets, as is conventional (e.g., 〈·〉).*

We have now replaced the less-than/greater-than symbols with angular brackets. Thanks for pointing this out.

*Reviewer #2: I have a few comments as follows:
1) The introduction provides useful information for the study. However, more information on the motivation for using the commute time measure, its biological plausibility, and comparison with other measures of signal transmission, including neural mass models, should be provided.*

Thank you for this important comment. We attempted to better highlight how commute time differentiates from previously proposed communication metrics, such as communicability, in the Introduction. We have also made an argument for its biological plausibility.

At the current stage of the work, commute time is not aimed to replace neural mass models. In other words, the goal of using commute time is not to quantitatively simulate brain function. Rather, the goal is to elucidate to what extent Markovian dynamics as calculated from the structural connectivity matrix, underly brain function. In the Results subsection “Commute captures simulated function”, we explore whether commute time can capture simulated functional connectivity from a neural mass model.

*2) The rationale for choosing only two communication measures (communicability and search information) was not clear. Other communication measures, such as mean first passage time, which are based on the diffusion process, should be considered for comparisons.*

Thank you for suggesting that we use mean first passage time as an additional metric. We have added a paragraph in the Results subsection “Commute time better captures brain function”, as well as corresponding Supplementary Figure S12 and Text S2 titled “Mean first passage time vs. hitting time” that explores mean first passage time as an alternative communication measure. To summarize, the mathematical expression for mean first passage time approximates hitting time, the component making up the commute time expression (Equation 1). Therefore, performances using mean first passage time are essentially the same as those for hitting time, when calculating commute time (Figure S12).

Below is the addition made to the text and the newly created Supplementary Figure S12. Please also see Supplementary Text S2 for the mathematical derivation of mean first passage time to understand its close relationship with hitting time (which exhibits a correlation coefficient of 0.90 as shown in the newly generated Fig S12B).

An alternative mathematical approach to calculate commute time is to express it in terms of mean first passage time, rather than hitting time. Mean first passage time is the large time limit approximation of hitting time (S2 Text). A previous study found mean first passage time to be one of the best FC correlates among 40 structure-based predictors (22). We also find that commute time as calculated by mean first passage time does just as well as when calculated by hitting time (Figure S12A) across UK Biobank individuals, because the two diffusion-based metrics are highly correlated (Figure S12B).

| **A** |  | **B** |  |
| --- | --- | --- | --- |

**Figure S12: Commute time calculated via hitting time or mean first passage time yield similar results. A.** demonstrates correlation results across all considered UK Biobank subjects; **B.** demonstrates how closely hitting time and mean first passage time are for an arbitrary UK Biobank subject across all possible pairs of its brain regions. fMRI and dMRI data are processed according to the Desikan-Killiany atlas.

*3) The analysis based on simulated FC was informative. However, have the authors considered including randomized FC/null models to check whether the observed relationships in simulated and actual FC are meaningfully constrained by the actual network architecture.*

We very much appreciate this suggestion and have now presented a brief discussion of the results from performing the null model in the Results: Commute captures simulated function. Figure S3A demonstrates the disappearance of the strong correlation as seen in Figure 2A for the null model.

*4) I may have missed this, but it seems that the statistics for correlation between the simulated functional connectivity and commute time are only reported at the individual level. It would strengthen the analysis to also provide population level statistics (e.g., group means, statistical significance).*

The statistics for the correlation between simulated functional connectivity and commute time as shown in Figure 2C is presented in Table S2. The Kolmogorov-Smirnov test is used which compares the distribution of groups. We do not explicitly report any statistics in the main text for Figure 2C’s results because the distributions between commute time and other structure-based metrics are so widely different. For other figures in the text, we do report some Kolmogorov-Smirnov test statistic values and their significances in the main text.

*5) The final section of the results, “Structure-function coupling strength weakly relates to biomarkers,” should be expanded. Specifically, it would be helpful to specify which brain diseases and mental disorders are included in the analysis. For each condition, more detailed information should be provided on how commute time is disrupted, such as the regional changes of the number of streamlines in disease condition and their correlation with the commute time.*

Thanks for this comment. We have now explicitly listed the names of the diseases included in the analysis in Figure 4 in Table S4. We have changed the name of the class ‘brain disease’ to ‘nerve disorder’ to better capture the diseases captured by International Classification of Diseases-10 code G.

As mentioned in the Results in the context of explaining aging results, having more streamlines trivially leads to a smaller commute time on average. This property is independent of an individual’s disease condition or age.

*Additionally, differences in functional and structural connectivity between disease and healthy groups should be reported to have a better context for alterations of structure-function coupling.*

We believe that this comment may already be captured by Figure 4C. There, we demonstrate distributions of the average commute time and average functional connectivity values across individuals in their respective disease group. We find that results presented in Figure 4A may be driven by large differences in the average FC values of individuals diagnosed with mental health disorders compared to the two other disease groups.
